# Comparative Analysis of Salivary Mycobiome Diversity in Human Immunodeficiency Virus-Infected Patients

**DOI:** 10.3389/fcimb.2021.781246

**Published:** 2021-12-01

**Authors:** Shenghua Chang, Haiying Guo, Jin Li, Yaoting Ji, Han Jiang, Lianguo Ruan, Minquan Du

**Affiliations:** ^1^ The State Key Laboratory Breeding Base of Basic Science of Stomatology (Hubei-MOST) and Key Laboratory of Oral Biomedicine Ministry of Education, School & Hospital of Stomatology, Wuhan University, Wuhan, China; ^2^ Department of Infectious Diseases Treatment, Wuhan Medical Treatment Center, Wuhan, China

**Keywords:** saliva, salivary mycobiome, human immunodeficiency virus, antiretroviral therapy, high-throughput sequencing

## Abstract

Reports on alterations in the oral mycobiome of HIV-infected patients are still limited. This study was designed to compare the salivary mycobiome between 30 human immunodeficiency virus (HIV) infections and 30 healthy controls and explore the effect of antiretroviral therapy (ART) administration on the oral mycobiome of HIV infections. Results showed that the diversity and richness of salivary mycobiome in HIV-infected individuals were higher than those of controls (*P* < 0.05). After ART, the diversity and richness of salivary mycobiome in HIV-infected patients were reduced significantly (*P* < 0.05). *Candida*, *Mortierella*, *Malassezia*, *Simplicillium*, and *Penicillium* were significantly enriched in the HIV group and dramatically decreased after ART. While the relative abundance of *Verticillium*, *Issatchenkia*, and *Alternaria* significantly increased in patients with HIV after ART. Correlation analysis revealed that *Mortierella*, *Malassezia*, *Simplicillium*, and *Chaetomium* were positively correlated with viral load (VL), whereas *Thyrostroma* and *Archaeorhizomyces* were negatively related to VL and positively related to CD4^+^ T-cell counts. All results showed that HIV infection and ART administration affected the composition of salivary mycobiome communities. Furthermore, differences of salivary mycobiome in HIV infections after ART were complex and might mirror the immune state of the body.

## Introduction

Human immunodeficiency virus (HIV) infection likely results in a progressive decrease in the number and function of CD4^+^ T lymphocytes; consequently, patients are susceptible to various opportunistic infections (Cohen et al., 2011). A high prevalence of HIV infection has been reported. There were approximately 37.6 million people living with HIV in the world at the end of 2020 and 1.5 million new infections in 2020 (Poole et al., 2013). Opportunistic infections are common among HIV-infected patients even in the era of antiretroviral therapy (ART) (Leigh et al., 2004; Aberg and Powderly, 2010). Candidiasis, pneumocystis pneumonia, and cryptococcal infections are frequently reported (Patil et al., 2018). Among them, oral candidiasis is the most common oral infection and could be detected in the early stage of HIV infection. Moreover, the risk of suffering from oral candidiasis by HIV-infected individuals, even those who have higher CD4^+^ T-cell counts, is higher than that by uninfected subjects (Owotade et al., 2008; Thompson et al., 2010; Hu et al., 2018); this finding can serve as a guidance for dentists to find a suspicious HIV infection (Armstrong-James et al., 2014). Oral candidiasis may also spread outside the mouth, causing candida infections in the esophagus or stomach (Hugh, 2014). Evidence has shown that similar mold and fungal communities are found in the respiratory tract, gastrointestinal tract, and mouth of HIV-infected individuals (Lijia et al., 2015; HC and GM, 2018). These findings suggested that the salivary mycobiome may play an important role in infectious diseases.

Saliva is a type of body fluid, which is nearly 50% similar to the blood, easy to collect and store (Pfaffe et al., 2011). Saliva contains human oral microorganisms, as well as DNA, RNA, proteins, and other parts, which makes it a good sample to provide information for clinical diagnosis (Zhang et al., 2016) in oral cancer (Park et al., 2009; Vidotto et al., 2010), diabetes mellitus (Abdolsamadi et al., 2014), cardiovascular disease (Zheng et al., 2014), and viral infections (Nefzi et al., 2015). Though there is a very diverse environment in saliva, the diversity of salivary microbiome is similar between individuals in short- and long-term longitudinal studies (Cameron et al., 2015; Belstrom et al., 2016; Wang et al., 2020).

In recent years, increasing studies have focused on the interaction between HIV infection and microorganisms. With the development of high-throughput sequencing technology, accumulating results have revealed significant alterations in the microbiome of the gastrointestinal tract (Ling et al., 2016), vagina (Cohen, 2016), lung (Twigg et al., 2017), and mouth (Kistler et al., 2015) in HIV-infected patients. Studies on the characteristics of the oral microbiome have also been widely performed, but most studies have focused on changes in oral bacteria. Besides, most of these studies are cross-sectional studies (Hegde et al., 2014; Li et al., 2014; Mukherjee et al., 2014; Beck et al., 2015; Kistler et al., 2015; Noguera-Julian et al., 2017), and only a few studies have investigated longitudinal variation in oral bacteria after ART administration (Navazesh et al., 2005; Li et al., 2014; Presti et al., 2018). Reports on alterations in the oral mycobiome of HIV-infected patients are still limited, and longitudinal variations are few.

In this study, next-generation sequencing (NGS) was conducted to analyze the characteristics of the salivary mycobiome in HIV-infected individuals and to further explore the variation in the salivary microbiome of HIV-infected patients after 6 months of ART administration.

## Methods

### Subject Recruitment

Newly HIV-infected patients were recruited from Wuhan Medical Treatment Center, China, and HIV-uninfected subjects were enrolled from the Wuhan University, China (this study was approved by the ethics committee of the School & Hospital of Stomatology, Wuhan University). HIV-infected participants were followed up for 6 months during ART administrations.

The inclusion criteria were as follows: 1) individuals were diagnosed with HIV in the past 1 year, without receiving antiretroviral therapy, or individuals were age- and gender-paired HIV-uninfected subjects; 2) participants aged over 18 years and under 60 years; 3) subjects who could cooperate and sign the informed consent forms.

The exclusion criteria were as follows: 1) subjects were diagnosed with HIV more than 1 year or with HIV in the past 1 year but received ART; 2) obvious clinical oral symptoms, including caries, periodontal disease, mucous disease, and oral surgical disease; 3) history of dental treatments in the past 6 months; 4) receiving antibiotics, immunomodulators, and probiotic treatments in the past 3 months; 5) history of infectious diseases, such as hepatitis B infection, tuberculosis infection, *Treponema pallidum* infection, and so on; 6) history of systemic diseases, such as diabetes, hypertension, and cancer; 7) history of inherited metabolic diseases and autoimmune diseases.

### Sample Collection

Subjects were instructed not to eat or drink within 1 h before sample collection. At rest, approximately 2 ml non-stimulated saliva was collected by using a 5-ml saliva collector (DNAgard^®^Saliva, Boykyo Pharmaceutical Co. Ltd., Tokyo, Japan). Then, 1.5 ml of preservation solution was added to saliva, and they were mixed upside down for 5 s. All samples were shipped to the laboratory on dry ice and stored in a refrigerator at -80°C for further use.

### Genomic DNA Isolation and PCR Amplification

The EZNATM Mag-Bind Soil DNA Kit (OMEGA M5635-02, Norcross, GA, USA) was used to extract DNA, and Illumina nest PCR was performed to amplify the full-length Internal Transcribed Spacer (ITS) gene. The first cycling conditions were as follows: initial denaturation at 94°C for 3 min, five cycles of denaturation at 94°C for 30 s, annealing at 45°C for 1 min, elongation at 65°C for 30 s, 20 cycles of denaturation at 94°C for 20 s, annealing at 55°C for 20 s, elongation at 72°C for 30 s, and a final extension step at 72°C for 5 min. Then, 20 ng of the PCR products of each sample were used for the second PCR amplification with specific primers (ITSF: CTTGGTCATTTAGAGGAAGTAA and ITS2R: GCTGCGTTCTTCATCGATGC). The second cycling conditions were as follows: initial denaturation at 94°C for 3 min, five cycles of denaturation at 94°C for 20 s, annealing at 55°C for 20 s, elongation at 72°C for 30 s, and a final extension step at 72°C for 5 min. The PCR products of the second amplification were purified using Agencourt AMPure XP (Transgen, EC401-03, Beckman Coulter, Brea, CA, USA) and then were accurately quantified with a Qubit 3.0 DNA detection kit (Life Q10210, Carlsbad, CA, USA). Lastly, 20 pmol of PCR products in the second amplification of each sample were used to sequence in Miseq2000 sequencing platform.

### Internal Transcribed Spacer rRNA Gene Sequence Analysis

The raw sequences obtained from this study were deposited into the NCBI Sequence Read Archive under the accession number PRJNA626395.

A total of 84 non-stimulated saliva samples were analyzed in this study. Prinseq (Schmieder and Edwards, 2011) was used to remove the bases with the read tail values less than 20 in each sample and cut out the N-containing sequences in reads. Then, short sequences were removed on the basis of the length threshold of 100 bp. After quality control (QC), 5,021,621 reads were obtained. The mean number of raw sequences was 59,781 (range: 35,983–78,391). The average sequence length was 240 bp. Filtered reads were processed with usearch (v.10.0) (Edgar and Flyvbjerg, 2015) in rstudio (v1.1.463) (Loraine et al., 2015). Unoise 3 (Edgar, 2018) was selected as the algorithm to remove the error results of PCR and sequencing and obtain zero-radius operational taxonomic units (zOTUs). Then, zOTUs were aligned to a reference database of known UNITE (Nilsson et al., 2015) for further analysis.

### Statistical Analysis

Chao1 and Shannon index were used to evaluate the richness and diversity of the salivary mycobiome in the three groups with the one-way analysis of variance (one-way ANOVA), and Tukey-Honestly significant difference (HSD) was used for pairwise comparisons between two groups. These results were visualized by using R vegan package (Oksanen J et al.). *P* < 0.05 was considered statistically different. The Bray–Curtis distance was selected to perform principal coordinates analysis (PCoA), and Adonis was used to assess the difference in shared diversity among the three groups. Besides, analysis of similarities (ANOSIM) was performed to test whether the difference between groups is significantly greater than the difference in the group. One-way ANOVA and Tukey-HSD were used to compare differential abundance taxa between groups. Linear discriminant analysis (LDA) effect size (LEfSe; http://huttenhower.sph.harvard.edu/galaxy/) was performed to explore the fungal biomarker in each group (LDA >2). All the samples in the three groups were fed into a random forest model to determine the contribution of the salivary mycobiome in classifying the groups. Correlative analysis with Spearman’s correlation coefficient was performed to analyze the relationship between the salivary mycobiome and CD4^+^ T-cell counts and viral load (VL) (Xiao et al., 2016).

## Results

### Subject Characteristics

Thirty newly HIV-infected men and 30 HIV-uninfected men were enrolled. In this study, 24 HIV-infected participants were followed up for 6 months during ART administrations. The mean ages in Control and HIV groups were 30.07 (range: 20–45) and 30.13 (range: 20–45) years, respectively. The mean CD4^+^ T-cell count in HIV group was 343.03 cells/µl (range: 20–642 cells/µl), and the mean VL count was 362,298 copies/ml (range: 867–8,749,628 copies/ml). After 6 months of ART administrations, the average of CD4^+^ T-cell counts in the HIV group increased to 456.92 cells/µl (range: 163–873 cells/µl), and the average VL count decreased to 121.75 copies/ml. The demographic and clinical characteristics of the participants are shown in **Supplementary Table 1**.

### Taxonomy Analysis

**Figure 1:**
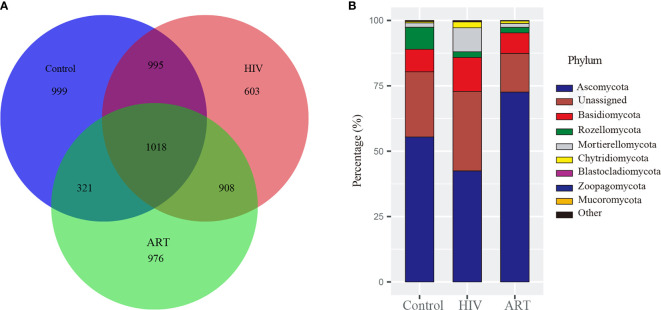
Taxonomy analysis of the salivary mycobiome structure among the Control, HIV, and antiretroviral therapy (ART) groups. **(A)** Venn diagram showed the number of zero-radius operational taxonomic units (zOTUs) among the three groups. **(B)** Bar chart described the phylum distribution of the salivary mycobiome among the three groups.

After the sequences were normalized sequences, 3,587, 3,333, and 3,223 zOTUs were obtained in HIV, Control, and ART groups, respectively (**Figure 1A**). There were 2,013 zOTUs detected in HIV and Control group and 1,339 zOTUs detected in Control and ART group, respectively. Moreover, 1,018 zOTUs were shared by the three groups. These results indicated that there were similarities and differences in the composition of salivary mycobiome among the three groups, but these results should be further statistically analyzed.

Then, taxonomic assignment was performed, and 11 phyla and 306 genera were obtained. The core mycobiome was contributed by five phyla: *Ascomycota*, *Basidiomycota*, *Rozellomycota*, *Mortierellomycota*, and *Chytridiomycota* (**Figure 1B**). In our study, *Aspergillus* and *Mortierella* could be detected from all the samples. In addition, there were three other genera with a detection rate of more than 90%, including *Penicillium* (96%), *Candida* (90%), and *Issatchenkia* (90%) (**Supplementary Figure 1** **)**.

### Alpha and Beta Diversity Analysis of the Salivary Mycobiome

**Figure 2:**
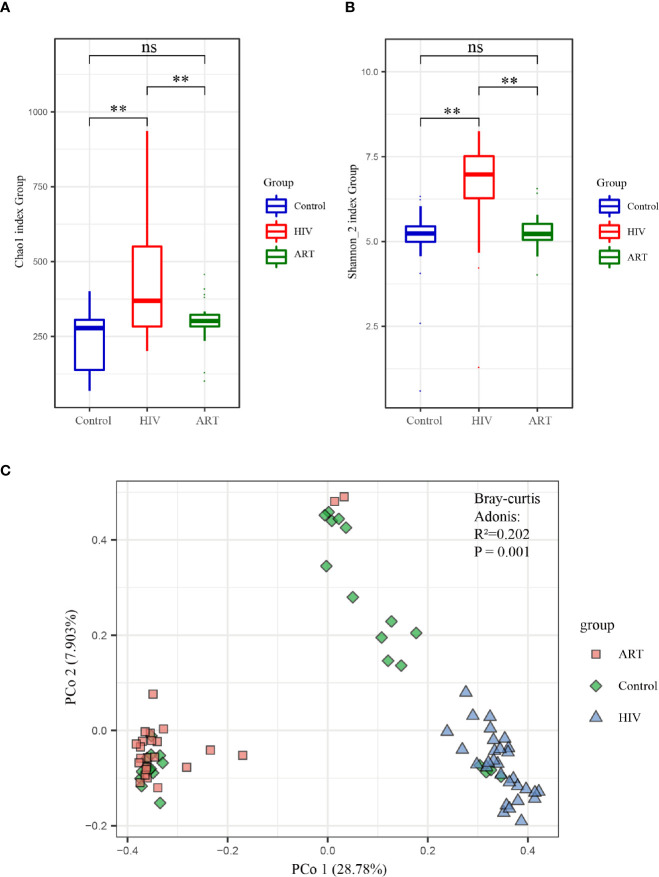
Alpha and beta diversity analyses of the salivary mycobiome among the three groups. **(A)** Chao1 index and **(B)** Shannon index showed the richness and evenness in the HIV, antiretroviral therapy (ART), and Control groups. **(C)** Principal coordinates analysis (PCoA) and Adonis analysis revealed the differences in the salivary mycobiome among the three groups. Blue represented the Control group, red indicated the HIV group, and green denoted the ART group. **P < 0.01; ns, no significant.

Alpha diversity analysis on the zOTU level revealed that the diversity and richness of the salivary mycobiome in the HIV group were higher than those in the Control group (*P* < 0.05) **(** **Figure 2A** **)**. After ART, the diversity and richness of the salivary mycobiome decreased significantly (*P* < 0.05) in HIV-infected individuals and were similar to those in the HIV-uninfected individuals (*P* > 0.05) **(** **Figure 2B** **)**.

PCoA and Adonis analysis were performed to further compare the community composition of the salivary mycobiome in the three groups. Both results revealed the differences in the mycobiome community composition among the groups (**Figure 2C**). Dramatically, the community composition of the HIV group was significantly different from that of the HIV group after 6 months of ART (ART group), but no differences were observed between the Control and ART groups (**Supplementary Table 2**). Consistent with the findings of alpha diversity analysis, these results suggested that ART did affect the community composition of the salivary mycobiome.

### Comparative Analysis of the Salivary Mycobiome Among the Three Groups

**Figure 3:**
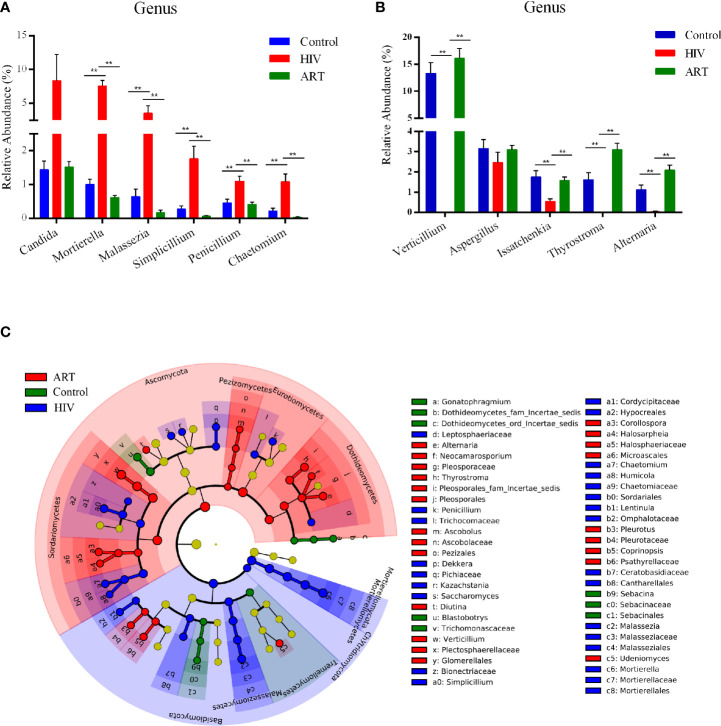
Taxon comparative analysis of the salivary mycobiome among the three groups at the genus level. **(A, B)** Histogram based on the results of Tukey-HSD showed the significantly different genera among groups. **P < 0.01, *P < 0.05 (mean ± SEM). **(C)** Linear discriminant analysis effect size (LEfSe) cladogram identified differently abundant taxa in each group. Red represented the antiretroviral therapy (ART) group-enriched taxa, blue indicated HIV group-enriched taxa, and green denoted Control group-enriched taxa.

At the phylum level, the abundance of *Basidiomycota*, *Mortierellomycota*, and *Chytridiomycota* was significantly higher in the HIV group than that in the Control and ART groups, and *Ascomycota* was more abundant in the ART group than that in the HIV and Control groups (**Supplementary Table 3** **)**. A total of 22 genera with a detection rate over 20% and relative abundance over 0.5% in one dominant group were selected to perform a comparative analysis (**Supplementary Table 4**). Among them, the relative abundance of *Mortierella*, *Malassezia*, *Simplicillium*, *Penicillium*, and *Chaetomium* was significantly higher in the HIV group than that in the other two groups (*P* < 0.05) (**Figure 3A**). Moreover, *Candida* was more abundant in the HIV group than in the Control and ART groups, although no statistically significant differences were observed (**Figure 3A**). In **Figure 3B**, the relative abundance of *Verticillium*, *Issatchenkia*, and *Alternaria* dramatically decreased in the HIV group compared with that in the Control group (*P* < 0.05). Interestingly, the three genera were significantly enriched again in the HIV-infected subjects after 6 months of ART (HIV group vs. ART group, *P* < 0.05, **Figure 3B**; results of the comparative analysis of 11 other genera are shown in **Supplementary Figure 2**). These statistical results were also supported by the results of LEfSe analysis performed in the three groups (**Figure 3C**). These findings showed that *Mortierella*, *Malassezia*, *Simplicillium*, *Penicillium*, and *Chaetomium* were sensitive to HIV infection, and *Verticillium* and *Alternaria* were sensitive to ART.

### Random Forest Analysis

**Figure 4:**
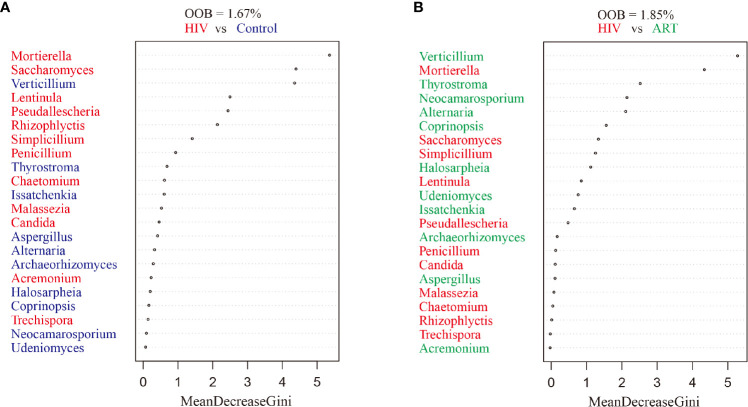
Random forest analysis. Random forest analysis classification of the ranked importance of the mycobiome for classifying the samples in HIV category (patients with and without HIV infection) **(A)** and in the antiretroviral therapy (ART) category (ART or not) **(B)**. OOB, out-of-box error rate.

Random forest classification was analyzed to further investigate the contribution of the salivary mycobiome in classifying HIV infection and ART administration. It revealed that the most important salivary mycobiome for categorizing HIV infection was *Mortierella* (**Figure 4A**), and *Verticillium* was the most important fungi to categorize ART state (**Figure 4B**), which was also in line with the results of the comparative analysis.

### Correlation Between the Salivary Mycobiome and CD4^+^ T-Cell Counts, Viral Load

**Figure 5:**
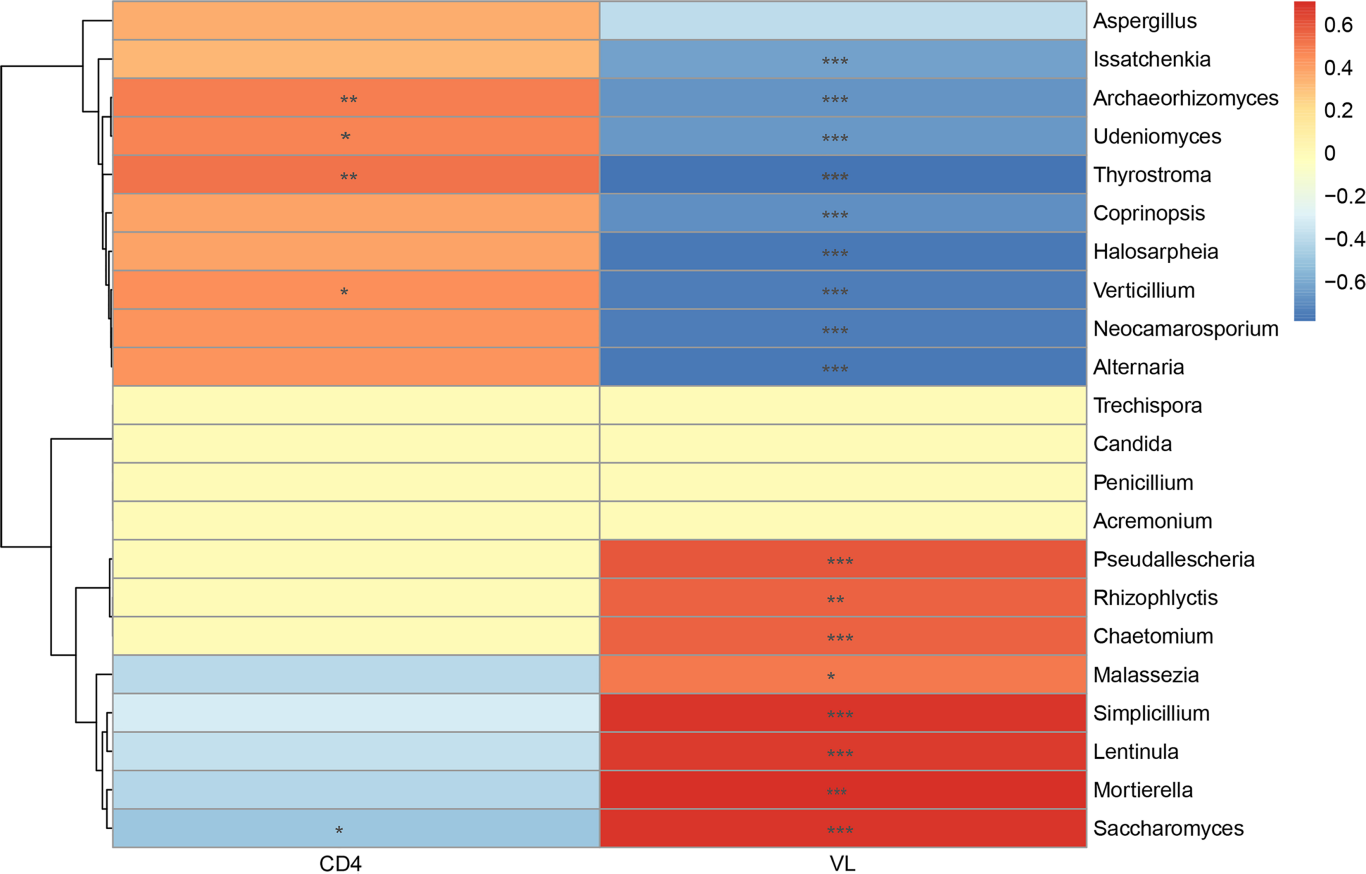
Correlation analysis between the salivary mycobiome and CD4^+^ T-cell counts and viral load (VL) in the blood. Diagram showed Spearman’s correlation coefficient (|rho| >0.3) with the *P <* 0.05. The significance are shown as flow: *P < 0.05, **P < 0.01, ***P < 0.001.

The increased CD4^+^ T-cell counts and decreased VL counts were predominant features of immunologic reconstitution in HIV-infected individuals after ART (Tiba et al., 2012). Correlation analysis by Spearman’s correlation coefficient was performed to further analyze the relationship between salivary mycobiome and CD4^+^ T-cell counts, VL counts (**Figure 5**). We found that *Mortierella*, *Malassezia*, and *Simplicillium* were positively correlated with VL, and *Verticillium*, *Alternaria*, and *Issatchenkia*etc were negatively correlated with VL. *Archaeorhizomyces* and *ThyrostromaIt* were positively correlated with CD4^+^ T-cell counts and negatively correlated with VL. It suggested that the community structure of saliva mycobiome of HIV-infected people had a relationship with CD4^+^ T-cell counts and VL in the blood. It suggested that the salivary mycobiome of HIV-infected people was related to CD4^+^ T-cell counts and VL in the blood.

## Discussion

Our study reported the differences in the composition of the salivary mycobiome between patients with HIV and HIV-uninfected subjects and the effect of 6 months of ART on the salivary mycobiomes.

Previous oral mycobiome studies mainly relied on traditional isolation and culture methods (Diaz et al., 2017). However, studies have shown that less than 1% of microorganisms, including fungi, can be cultivated under laboratory conditions (Rappe and Giovannoni, 2003). Therefore, using traditional isolation and culture methods to study the relationship between mycobiome and diseases may leave some biases. Microbial detection methods based on molecular biology techniques have emerged and expanded our understanding of oral mycobiome (Chiu and Miller, 2019).

In 2010, Ghannoum et al. (2010) used 454 pyrosequencing to analyze oral flushing samples from 20 healthy individuals and found 101 fungal genera, including 11 non-cultivable genera. Four common pathogenic fungi, including *Candida*, *Aspergillus*, *Fusarium*, and *Cryptococcus* were detected in more than 20% of subjects in this study, and these mycobiomes were also detected in our study. Apart from these fungi, *Malassezia* was a common fungus in the oral mycobiome of healthy subjects in another study (Dupuy et al., 2014). This finding was consistent with our result, too.

Mukherjee et al. (2014) used the 454 pyrosequencing to compare the oral core fungal flora of 12 HIV-infected patients and 12 HIV-uninfected individuals and found that the oral core fungi were different between the two groups (the “core” flora were microorganisms detected in more than 20% of the subjects). Interestingly, *Candida* and *Penicillium* were common in the two groups in this study. While *Aspergillus* and *Mortierella* were detected in all the samples in our study. Besides, the frequency of *Candida*, *Epicoccum*, and *Alternaria* was much higher in the HIV group than that in the healthy group (Mukherjee et al., 2014), but the relative abundance of *Mortierella*, *Malassezia*, and *Penicillium* was much higher in the HIV group than that in the other group in our study. Dramatically, *Alternaria* was enriched in the control group in our study. Furthermore, only two of 22 dominant genera were not significantly different in our comparison analysis. Some differences were observed between our study and the research conducted by Mukherjee et al. (2014) because of the following: 1) the race and age of the included subjects were different; 2) the sequencing methods were different; 3) the samples were different, that is, mouth wash samples and non-stimulated whole saliva samples were selected in the two studies; 4) most of all, the characteristics of the included HIV-infected subjects were different. HIV-infected patients were newly infected within 1 past year in our study, and the body’s immune system might not be severely damaged at this moment; as such, patients could not easily suffer from secondary infection. This condition was the main reason why the most common infecting genus in patients with HIV—*Candida*—was not more enriched in HIV group compared with Control group in our study.

In addition, we explored the effect of ART on the composition of the salivary mycobiome and found that the richness and diversity of salivary mycobiome decreased after ART administration. Moreover, the composition of the salivary mycobiome in the ART group was similar to that in the Control group. Furthermore, *Verticillium*, *Archaeorhizomyces*, and *Thyrostroma* were negatively correlated with VL and positively correlated with CD4^+^ T-cell counts. By comparison, *Saccharomyces* was positively correlated with VL and negatively correlated with CD4^+^ T-cell counts. These results indicated that ART administration could affect the composition of the salivary mycobiome in HIV-infected patients, and some fungi were sensitive to the changes in CD4^+^ T-cell counts and VL in the blood.

Our study had some limitations. First, the saliva samples collected from the subjects before acquiring HIV were the best control. This study was limited by ethics and difficulties in collecting the samples, so saliva was obtained from HIV-uninfected subjects as the control. Second, the follow-up period was cut short because of coronavirus disease 2019 (COVID-19). Third, the sample size was not large enough. Fourth, many sequences could not be classified and annotated with the incomplete fungal ribosome database. Fifth, specific species were not detected because of the difficulties in generating similarity thresholds to define species-level operable taxonomic units. Therefore, more cohort studies with a long follow-up period and a large sample size should be performed to further analyze the characteristics of the salivary mycobiome in HIV-infected subjects. Besides, more research is needed to improve the fungal ribosome database that should be completed. Standard methods to explore human fungal microorganisms also need to be improved.

## Conclusions

In this study, we found that HIV infection and ART administration might affect the composition of the salivary mycobiome. Furthermore, differences in the salivary mycobiome in HIV infections after ART were complex and might mirror the immune state of the body. In the future, studies should be performed on the salivary mycobiome with a large sample size and long follow-up time.

## Data Availability Statement

The datasets presented in this study can be found in online repositories. The names of the repository/repositories and accession number(s) can be found below: https://www.ncbi.nlm.nih.gov/, PRJNA626395.

## Ethics Statement

The studies involving human participants were reviewed and approved by the ethics committee of the School & Hospital of Stomatology, Wuhan University. The patients/participants provided their written informed consent to participate in this study.

## Author Contributions

The study was conceptualized and designed by MD, SC, HG, and JL. SC and HG performed the experiments. SC analyzed the data and made figures for the article. SC and HG wrote the original draft of the article. HG, JL, YJ, HJ, and MD reviewed and revised the article. LR was the expert on HIV/AIDS, who provided and evaluated the samples of HIV infections. All authors contributed to the article and approved the submitted version.

## Funding

This study was supported by the National Natural Science Foundation of China (No. 81771084).

## Conflict of Interest

The authors declare that the research was conducted in the absence of any commercial or financial relationships that could be construed as a potential conflict of interest.

## Publisher’s Note

All claims expressed in this article are solely those of the authors and do not necessarily represent those of their affiliated organizations, or those of the publisher, the editors and the reviewers. Any product that may be evaluated in this article, or claim that may be made by its manufacturer, is not guaranteed or endorsed by the publisher.
