# Supplementary material for: Comparative Analysis of Salivary Mycobiome Diversity in Human Immunodeficiency Virus-Infected Patients
Source: Front Cell Infect Microbiol. 2021 Dec 1;11:781246. doi: 10.3389/fcimb.2021.781246 (PMC8671614; doi:10.3389/fcimb.2021.781246)
Supplement: Supplementary file 1 [file DataSheet_1.docx]

Supplementary Material

# Table 1 Demographic and clinical characteristics of the participants

|  | HIV  (n=30) | Control  (n=30) | ART  (n=24) | P-Value |
| --- | --- | --- | --- | --- |
| Age (year) | 30.13±7.30 | 30.07±7.29 | 31.83±6.92 | 0.440 |
| Gender [N (%)] |  |  |  | - |
| Male | 30(100%) | 30(100%) | 24(100%) | - |
| Female | 0 | 0 | 0 | - |
| CD4⁺T cell counts(cells/ul) | 343.03±154.57 | - | 456.92±190.54 | 0.006 |
| VL (copies/ml) | 362,298.00±1,587,904.84 | - | 121.75±435.76 | 0.542 |

Note: CD4 + T cells counts and VL counts, the data comes from Medical Treatment Center, Wuhan, China

# Table 2 ANOSIM analysis with Bray-curtis

| Group | distance | R | P_value |
| --- | --- | --- | --- |
| HIV & Control | Bray-Curtis | 0.646 | 0.001 |
| HIV & ART | Bray-Curtis | 0.929 | 0.001 |
| ART & Control | Bray-Curtis | 0.019 | 0.186 |

# Table 3 The outcomes of comparative analysis of salivary mycobiome in phylum level between groups

| Variable | (I) Group | (J) Group | Mean Difference (I-J) | Std.Err | Sig. | 95% Confidence Interval | |
| --- | --- | --- | --- | --- | --- | --- | --- |
|  |  |  |  |  |  | Lower bound | Upper bound |
| *Ascomycota* | HIV | Control | -12.98100^*^ | 4.63012 | .017 | -24.0356 | -1.9264 |
|  |  | ART | -30.14333^*^ | 4.91099 | .000 | -41.8685 | -18.4181 |
|  | ART | Control | 17.16233^*^ | 4.91099 | .002 | 5.4371 | 28.8875 |
| *Basidiomycota* | HIV | Control | 4.35730^*^ | 1.33914 | .005 | 1.1601 | 7.5545 |
|  |  | ART | 5.09397^*^ | 1.42037 | .002 | 1.7028 | 8.4852 |
|  | ART | Control | -.73667 | 1.42037 | .862 | -4.1279 | 2.6545 |
| *Rozellomycota* | HIV | Control | -6.22298 | 2.62044 | .052 | -12.4794 | .0334 |
|  |  | ART | .08756 | 2.77940 | .999 | -6.5484 | 6.7235 |
|  | ART | Control | -6.31054 | 2.77940 | .066 | -12.9465 | .3254 |
| *Mortierellomycota* | HIV | Control | 7.48180^*^ | .80707 | .000 | 5.5549 | 9.4087 |
|  |  | ART | 7.64129^*^ | .85603 | .000 | 5.5975 | 9.6851 |
|  | ART | Control | -.15949 | .85603 | .981 | -2.2033 | 1.8843 |
| *Chytridiomycota* | HIV | Control | 1.75583^*^ | .28168 | .000 | 1.0833 | 2.4283 |
|  |  | ART | 1.39845^*^ | .29876 | .000 | .6851 | 2.1118 |
|  | ART | Control | .35739 | .29876 | .459 | -.3559 | 1.0707 |
| * The mean difference is significant at the 0.05 level. | | | | | | | |

**Table 4** The outcomes of comparative analysis of salivary mycobiome in genus level between groups

| Variable | (I) Group | (J) Group | Mean Difference (I-J) | Std.Err | Sig. | 95% Confidence Interval | |
| --- | --- | --- | --- | --- | --- | --- | --- |
|  |  |  |  |  |  | Lower bound | Upper bound |
| *Mortierella* | HIV | Control | 6.55331^*^ | .71701 | .000 | 4.8414 | 8.2652 |
|  |  | ART | 6.94392^*^ | .76050 | .000 | 5.1282 | 8.7597 |
|  | ART | Control | -.39061 | .76050 | .865 | -2.2064 | 1.4251 |
| *Archaeorhizomyces* | HIV | Control | -.53609^*^ | .14543 | .001 | -.8833 | -.1889 |
|  |  | ART | -.43020^*^ | .15426 | .018 | -.7985 | -.0619 |
|  | ART | Control | -.10589 | .15426 | .772 | -.4742 | .2624 |
| *Penicillium* | HIV | Control | .64671^*^ | .16048 | .000 | .2636 | 1.0299 |
|  |  | ART | .68408^*^ | .17022 | .000 | .2777 | 1.0905 |
|  | ART | Control | -.03737 | .17022 | .974 | -.4438 | .3690 |
| *Aspergillus* | HIV | Control | -.70229 | .58615 | .458 | -2.1018 | .6972 |
|  |  | ART | -.65692 | .62171 | .544 | -2.1413 | .8274 |
|  | ART | Control | -.04537 | .62171 | .997 | -1.5297 | 1.4390 |
| *Udeniomyces* | HIV | Control | -.56324^*^ | .14825 | .001 | -.9172 | -.2093 |
|  |  | ART | -.97740^*^ | .15725 | .000 | -1.3528 | -.6020 |
|  | ART | Control | .41416^*^ | .15725 | .027 | .0387 | .7896 |
| *Acremonium* | HIV | Control | .36888 | .29091 | .417 | -.3257 | 1.0634 |
|  |  | ART | -.34725 | .30856 | .501 | -1.0839 | .3895 |
|  | ART | Control | .71612 | .30856 | .059 | -.0206 | 1.4528 |
| *Issatchenkia* | HIV | Control | -1.20616^*^ | .30563 | .000 | -1.9359 | -.4765 |
|  |  | ART | -1.03723^*^ | .32417 | .006 | -1.8112 | -.2633 |
|  | ART | Control | -.16893 | .32417 | .861 | -.9429 | .6050 |
| *Rhizophlyctis* | HIV | Control | .64473^*^ | .11167 | .000 | .3781 | .9113 |
|  |  | ART | .62687^*^ | .11844 | .000 | .3441 | .9097 |
|  | ART | Control | .01787 | .11844 | .988 | -.2649 | .3007 |
| *Pseudallescheria* | HIV | Control | .63853^*^ | .12793 | .000 | .3331 | .9440 |
|  |  | ART | .65654^*^ | .13569 | .000 | .3326 | .9805 |
|  | ART | Control | -.01801 | .13569 | .990 | -.3420 | .3060 |
| *Malassezia* | HIV | Control | 2.90206^*^ | .94005 | .008 | .6576 | 5.1465 |
|  |  | ART | 3.37125^*^ | .99708 | .003 | .9907 | 5.7518 |
|  | ART | Control | -.46920 | .99708 | .885 | -2.8498 | 1.9114 |
| *Candida* | HIV | Control | 6.93833 | 3.28450 | .094 | -.9036 | 14.7802 |
|  |  | ART | 6.85602 | 3.48374 | .127 | -1.4616 | 15.1736 |
|  | ART | Control | .08231 | 3.48374 | 1.000 | -8.2353 | 8.3999 |
| *Chaetomium* | HIV | Control | .86261^*^ | .21118 | .000 | .3584 | 1.3668 |
|  |  | ART | 1.04764^*^ | .22399 | .000 | .5128 | 1.5824 |
|  | ART | Control | -.18502 | .22399 | .688 | -.7198 | .3498 |
| *Trechispora* | HIV | Control | .45798^*^ | .18154 | .036 | .0246 | .8914 |
|  |  | ART | .46842^*^ | .19255 | .045 | .0087 | .9281 |
|  | ART | Control | -.01044 | .19255 | .998 | -.4702 | .4493 |
| *Neocamarosporium* | HIV | Control | -.40370^*^ | .11051 | .001 | -.6676 | -.1398 |
|  |  | ART | -.72681^*^ | .11722 | .000 | -1.0067 | -.4470 |
|  | ART | Control | .32311^*^ | .11722 | .020 | .0433 | .6030 |
| *Simplicillium* | HIV | Control | 1.49385^*^ | .32183 | .000 | .7255 | 2.2622 |
|  |  | ART | 1.70325^*^ | .34135 | .000 | .8883 | 2.5182 |
|  | ART | Control | -.20940 | .34135 | .813 | -1.0244 | .6056 |
| *Alternaria* | HIV | Control | -1.08320^*^ | .25742 | .000 | -1.6978 | -.4686 |
|  |  | ART | -2.05181^*^ | .27304 | .000 | -2.7037 | -1.3999 |
|  | ART | Control | .96861^*^ | .27304 | .002 | .3167 | 1.6205 |
| *Coprinopsis* | HIV | Control | -.51341^*^ | .15763 | .005 | -.8898 | -.1371 |
|  |  | ART | -.87929^*^ | .16719 | .000 | -1.2785 | -.4801 |
|  | ART | Control | .36588 | .16719 | .079 | -.0333 | .7651 |
| *Lentinula* | HIV | Control | .68781^*^ | .11727 | .000 | .4078 | .9678 |
|  |  | ART | .71189^*^ | .12438 | .000 | .4149 | 1.0089 |
|  | ART | Control | -.02408 | .12438 | .980 | -.3210 | .2729 |
| *Halosarpheia* | HIV | Control | -.30807^*^ | .08707 | .002 | -.5160 | -.1002 |
|  |  | ART | -.52073^*^ | .09235 | .000 | -.7412 | -.3002 |
|  | ART | Control | .21266 | .09235 | .061 | -.0078 | .4332 |
| *Thyrostroma* | HIV | Control | -1.61217^*^ | .35377 | .000 | -2.4568 | -.7675 |
|  |  | ART | -3.10083^*^ | .37523 | .000 | -3.9967 | -2.2050 |
|  | ART | Control | 1.48867^*^ | .37523 | .000 | .5928 | 2.3845 |
| *Saccharomyces* | HIV | Control | .65662^*^ | .14801 | .000 | .3032 | 1.0100 |
|  |  | ART | .66061^*^ | .15699 | .000 | .2858 | 1.0354 |
|  | ART | Control | -.00399 | .15699 | 1.000 | -.3788 | .3708 |
| *Verticillium* | HIV | Control | -13.32300^*^ | 2.06924 | .000 | -18.2634 | -8.3826 |
|  |  | ART | -16.10458^*^ | 2.19476 | .000 | -21.3447 | -10.8645 |
|  | ART | Control | 2.78158 | 2.19476 | .418 | -2.4585 | 8.0217 |
| *. The mean difference is significant at the 0.05 level. | | | | | | | |





**Figure 1** The frequency of genera in three groups.

**

**

**Figure 2** Taxa comparative analysis in genus level showed more abundant genera of HIV group (a) and Control, ART group (b). Turkey_HSD was used to multiple comparisons between groups, ***P* < 0.01, **P*< 0.05 (Mean ±SEM)

**
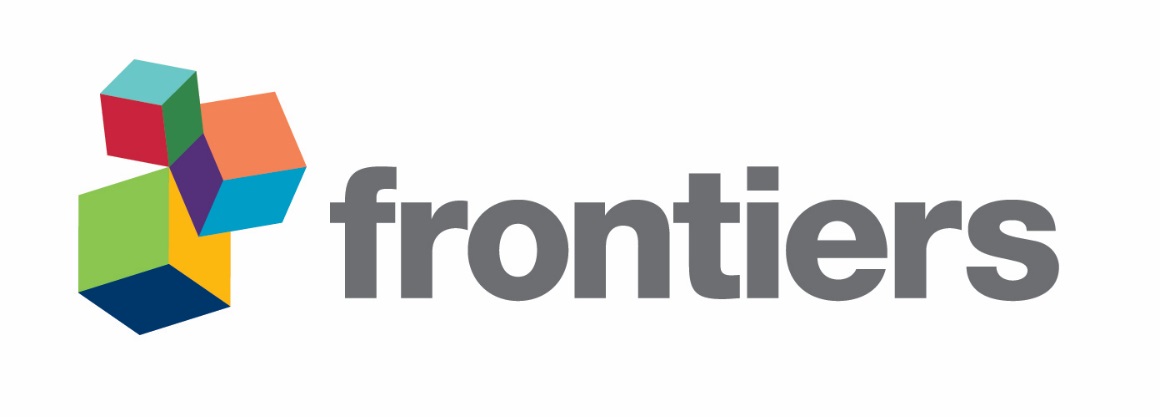
**
